# ASO Author Reflections: The Effects of Perioperative Corticosteroids on Postoperative Complications After Pancreatoduodenectomy: A Debated Topic of Systematic Review and Meta-analysis

**DOI:** 10.1245/s10434-024-16831-3

**Published:** 2025-01-24

**Authors:** Haonan Liu, Kongyuan Wei, Zheng Wu

**Affiliations:** 1 Department of Hepatobiliary Surgery, The First Affiliated Hospital of Xi’an Jiaotong University, Xi’an, Shaanxi China; 2 Pancreas Center, Xi’an Jiaotong University, Xi’an, Shaanxi China

## Abstract

**Introduction:**

Pancreatoduodenectomy (PD) is a complex surgery with high morbidity and mortality, often associated with complications like post-pancreatectomy hemorrhage (PPH) and postoperative pancreatic fistulas (POPF). The corticosteroids administered intraoperatively has been shown to improve postoperative outcomes in patients undergoing surgery. However, their impact on complications following PD remains controversial.

**Results:**

A comprehensive review of literature found no significant impact of perioperative corticosteroids on postoperative complications in PD, including postoperative major complications (PMCs), POPF, infectious complications, delayed gastric emptying (DGE), post-pancreatectomy hemorrhage (PPH), bile leakage, reoperation, or 30-days mortality.

**Conclusion:**

Other medications, such as octreotide, antibiotics, and probiotics, have shown potential in reducing complications, but further research is needed, especially for corticosteroids in PD. In the near future, more randomized controlled trials should be conducted, along with well-designed clinical studies, to provide high-level evidence for the potential benefits of corticosteroids in PD for targeted populations.

Pancreatoduodenectomy (PD) is considered one of the most challenging and complex surgical procedures, associated with a high incidence of adverse events. Dexamethasone has been approved as the standard therapy for the prevention and treatment of postoperative nausea and vomiting (PONV). Some studies have shown that the administration of corticosteroids during surgery can improve the postoperative prognosis in surgical patients and reduce the incidence of postoperative complications.^1^ Given the anti-inflammatory and immunosuppressive properties of corticosteroids, and considering the complexity and specificity of pancreatic surgery, which includes PD, a procedure that is particularly intricate, high-risk, with a high postoperative morbidity and mortality rate, and associated with complications, such as post-pancreatectomy hemorrhage (PPH) and postoperative pancreatic fistulas (POPF), corticosteroids are not widely accepted by pancreatic surgeons for its application in PD.^2^ In recent years, some retrospective studies have assessed the potential of perioperative corticosteroids in reducing postoperative complications in PD patients by comparing the short- and long-term outcomes between patients receiving corticosteroids and those who did not. However, the impact of perioperative corticosteroid administration on postoperative complications in PD remains controversial. To date, there is no systematic review or meta-analysis. This study was designed to provide a comprehensive review of the existing literature to evaluate the specific effects of perioperative corticosteroids on postoperative complications in PD patients, with the intention of offering some reference for the application of perioperative corticosteroids in the context of PD.^3^

According to the available literature, the results demonstrated that the use of corticosteroids in PD had no impact on the risk of postoperative complications. Specifically, we concluded that perioperative corticosteroids did not significantly demonstrate any distinction in terms of postoperative complications after PD, covering postoperative major complications (PMCs), POPF, infectious complications, delayed gastric emptying (DGE), PPH, bile leakage, reoperation, or 30-day mortality. This indicated that corticosteroids cannot be applied in the perioperative period to prevent postoperative complications of PD on account of existing results, but it may provide some reference for the prevention of PONV after pancreatic surgery.

The incidence of postoperative complications is very high in patients undergoing pancreatic surgery, and then perioperative medication is very indispensable to prevent postoperative complications. Postoperative nausea and vomiting is a common adverse reaction after anesthesia and surgery. In a grid meta-analysis, Weibel et al.^4^ compared 44 single drugs and 51 combination drugs in abdominal surgery and found that five single drugs (aprepitant, ramosetron, granisetron, dexamethasone, and ondansetron) reduced vomiting, and the drug in combination was better than the drug alone. Postoperative pancreatic fistulas is the most serious and recurrent complication after pancreatic surgery. A meta-analysis conducted by Chunli Wang concluded that perioperative prophylactic octreotide is effective in preventing pancreatic postoperative complications, especially the occurrence of POPF and effusion, as well as the occurrence of postoperative pancreatitis. However, octreotide did not significantly improve mortality. The occurrence rate of postoperative infection in PD is also extremely high, and bile contamination is known to be related to surgical site infection. Hence, the use of antibiotics in perioperative period to prevent postoperative complications has also been reviewed in many literatures. A clinical trial comparing the effects of piperacillin-tazobactam and cefoxitin in perioperative prevention of PD found that piperacillin-tazobactam can efficiently decrease infection and POPF, and it may be mediated by cefoxitin resistant biliary pathogens, especially enterobacterium and enterococcus.^5^ A single-center retrospective study also validated the use of piperacillin-tazobactam as perioperative prophylaxis in patients with PD to significantly reduce organ/space infections. There are also some other drugs that have been proven to reduce postoperative complications, such as some probiotics and prebiotics, which have the ability to protect the intestinal barrier, prevent bacterial migration, infection and postoperative complications. Studies have shown that perioperative administration of probiotics and prebiotics can reduce postoperative mortality and complication rate of patients undergoing surgery for periampullary neoplasms. However, its influence on pancreatic surgery remains to be further studied. Dexamethasone, as a steroid drug with different biological effects from other drugs, can not only prevent PONV, but also reduce postoperative inflammation by inhibiting the cycloxygenase pathway, stabilizing the neuron cell membrane and reducing the level of bradykinin in the tissue, thereby reducing postoperative pain. Dexamethasone has also been shown to inhibit the release of neuropeptides, such as calcitonin gene-related peptides and substance P, from nerve endings after tissue injury. A single administration can suppress cortisol concentrations for up to a week, and corticosteroids, such as dexamethasone, can prevent the body’s excessive stress response and may promote postoperative recovery.

According to the latest Chinese expert consensus on the perioperative use of corticosteroids, there are no clear recommendations on the scope of perioperative corticosteroids use, which are still widely used for preventing PONV and assisting analgesia. Regarding its application in pancreatic surgery, especially in PD, a consensus has not yet been reached. And concerning the impact of corticosteroids on postoperative complications, the guidelines do not provide a detailed explanation. At present, different preoperative drugs are used in different centers to prevent postoperative complications. This study may provide new evidence in guidelines for the appropriate management of preoperative prophylactic drugs. In the near future, more randomized controlled trials should be conducted, along with well-designed clinical studies, to provide high-level evidence for the potential benefits of corticosteroids in PD for targeted populations. Future research may seek to identify populations that benefit from the application of perioperative corticosteroids in postoperative outcomes following PD, thereby providing a more reliable reference for the use of corticosteroids in PD surgery.

Regarding basic research, the effects of corticosteroids on pancreatic neoplasms remain unknown. In the future, the role of corticosteroids in PD surgery is worthy of further large-scale trial exploration.
